# Lower Choline and Myo-Inositol in Temporo-Parietal Cortex Is Associated With Apathy in Amnestic MCI

**DOI:** 10.3389/fnagi.2018.00106

**Published:** 2018-04-13

**Authors:** Shankar Tumati, Esther M. Opmeer, Jan-Bernard C. Marsman, Sander Martens, Fransje E. Reesink, Peter P. De Deyn, André Aleman

**Affiliations:** ^1^Department of Neuroscience, University of Groningen, University Medical Center Groningen, Groningen, Netherlands; ^2^Mind, Brain Imaging and Neuroethics Research Unit, Institute of Mental Health Research, University of Ottawa; Royal Ottawa Mental Health Centre, Ottawa, ON, Canada; ^3^Department of Neurology, University of Groningen, University Medical Center Groningen, Groningen, Netherlands; ^4^Laboratory of Neurochemistry and Behavior, Institute Born-Bunge, University of Antwerp, Antwerp, Belgium; ^5^Department of Psychology, University of Groningen, Groningen, Netherlands

**Keywords:** apathy, amnestic MCI, temporo-parietal cortex, goal-directed behavior, choline, myo-inositol, MRS

## Abstract

Apathy is a common symptom in patients with amnestic mild cognitive impairment (aMCI) and is associated with an increased risk of progression to Alzheimer’s disease (AD). The neural substrates underlying apathy in aMCI may involve multiple brain regions, including the anterior cingulate cortex and the temporo-parietal region. Here we investigated neurometabolites in brain regions that may underlie apathy in aMCI patients using proton magnetic resonance spectroscopy (^1^H-MRS). Twenty-eight aMCI patients with varying degrees of apathy and 20 matched controls underwent ^1^H-MRS. Spectra were acquired from single voxels in the posterior cingulate cortex (PCC), dorsal anterior cingulate cortex (DACC), right dorsolateral prefrontal cortex (DLPFC), and right temporo-parietal cortex (TPC). Apathy was measured with the Apathy Evaluation Scale (AES). Spearman partial correlations between metabolite concentrations in each region and severity of apathy were determined. Additionally, analyses of covariance (ANCOVA) were performed to determine whether metabolite changes differed between patients with or without clinically-diagnosed apathy. The degree of apathy was found to be negatively correlated with choline and myo-inositol (mI) in the TPC. Additional exploratory analyses suggested that N-acetylaspartate (NAA)/mI ratio was reduced in aMCI without clinical apathy but not in aMCI with clinical apathy. In the DACC, glutamate and glutamine (Glx) levels tended to be higher in the aMCI with apathy group compared to controls and reduced in association with depression scores. In conclusion, apathy in aMCI patients was associated with neurometabolite changes indicative of altered membranal integrity and glial function in the right TPC. Findings also indicated that in a clinically-diagnosed aMCI cohort, apathy symptoms may be suggestive of neural changes that are distinct from aMCI without apathy.

## Introduction

In aMCI and AD dementia, apathy is associated with an increased risk for disease progression and poor functional outcomes (Boyle et al., 2003; Teng et al., 2007; Lanctôt et al., 2016). Characterized by reduced initiative and interest in activities (Robert et al., 2009), apathy is the most common neuropsychiatric syndrome in MCI and AD, and increases in incidence and severity with disease progression (Lyketsos et al., 2002; Geda et al., 2008; Zhao et al., 2016). Moreover, in MCI patients, impairment in more than one cognitive domain was associated with increased severity of apathy (Di Iulio et al., 2010). Symptoms of apathy have also been shown to be comorbid with depression and the symptoms of the two syndromes overlap (Marin et al., 1993; Tagariello et al., 2009). Unraveling the two syndromes, previous studies have shown that apathy is associated with a higher risk of functional impairment and disease progression independent of depression (Vicini Chilovi et al., 2009; Palmer et al., 2010; Richard et al., 2012).

Brain imaging studies have associated apathy in AD and aMCI with structural, functional, and metabolic changes in the DACC and the TPC (Stella et al., 2014; Kos et al., 2016). In particular, atrophy in the lateral temporal cortex in patients with aMCI was associated with the severity of apathy concurrently or with future development of apathy (Donovan et al., 2014). However, a previous study did not find this association (Zahodne et al., 2013). In these patients, impaired functional connectivity in the fronto-parietal network has also been reported (Munro et al., 2015). Moreover, sub-clinical apathy in healthy subjects is associated with changes in WM (Spalletta et al., 2013) as well as task-related functional magnetic resonance imaging (MRI) activation (Klaasen et al., 2017). These multimodal reports suggest the likely regions underlying apathy. However, the neurochemical changes, which have been well-characterized in aMCI and AD (Kantarci et al., 2013), associated with apathy are not known.

Studies in aMCI and AD patients using ^1^H-MRS (Alger, 2010) have consistently found reduced NAA, increased mI, and in some studies, increased choline in the PCC (Tumati et al., 2013). These neurochemical changes are also associated with cerebrospinal fluid and neuropathological markers of AD (Gomar et al., 2014; Murray et al., 2014). Based on these findings, a reduced ratio of NAA/mI has been suggested as a marker of AD and MCI. In the current study we sought to determine metabolite changes that may be present in the various regions implicated in apathy – the DACC and TPC, which were empirically associated with apathy (Stella et al., 2014; Kos et al., 2016); the DLPFC, as suggested by theoretical models of apathy (Levy and Dubois, 2006); and the PCC, where metabolite changes were expected in aMCI. We hypothesized that metabolite changes in the DACC and TPC would be associated with apathy, in accordance with existing literature. Specifically, reduced NAA/mI in the PCC and increased Cho and mI in the DACC and right TPC were hypothesized to be associated with greater apathy.

## Materials and Methods

### Participants

The study was conducted at the Neuroimaging Center, the Department of Geriatrics, and Alzheimer Research Center of the University Medical Center Groningen (UMCG), Netherlands. The study protocol was approved by the Medical Ethics Committee of the UMCG. Written informed consent was obtained from all study participants in accordance with the Declaration of Helsinki. In total, twenty-eight subjects with aMCI and 20 healthy cognitively normal subjects between 60 to 80 years of age were included in this study. They were all assessed by a neurologist and neuropsychologist for aMCI according to criteria by Petersen et al. (1999). Apathy was diagnosed clinically in eight subjects according to criteria described by Robert et al. (2009) and its severity was assessed with the AES (Clarke et al., 2007). The cognitively healthy subjects were matched with the aMCI group for age, gender and education, were required to have a MMSE score of 28 or higher, and to not have subjective or objective memory complaints. Subjects were excluded if (i) any neurological or psychiatric disorders (except symptoms of depression in aMCI subjects) were present currently or in the past, (ii) medications that may affect cognition were being used, (iii) a history of head injury accompanied by loss of consciousness was present, (iv) they were unable to undergo a MRI scan, or (v) anatomical abnormalities were found on the MRI scan. Two subjects with aMCI did not complete the questionnaire to assess depression and hence were excluded from the analyses, leaving a sample of 26 aMCI subjects and 20 controls.

### Cognitive and Behavioral Assessment

All subjects were evaluated with the MMSE, Global Deterioration scale, 30-item GDS (Yesavage, 1988) and the clinician version of the AES (Clarke et al., 2007). The AES is an 18-item ordinal scale, where each item is scored between 1 and 4 based on the clinician’s assessment. The assessment yields a score ranging from 18 to 72, with higher scores indicating greater apathy (Clarke et al., 2007).

All subjects also underwent cognitive testing for memory performance (15-word Rey’s Auditory Verbal Learning Test – immediate and ∼30 min delayed recall), processing speed (Digit symbol substitution test, and Trail making test part A), confrontational naming (Boston Naming test), and executive functions (Digit span forward and backward, Trail making test part B, and Hayling test). On these tests, aMCI subjects showed reduced memory performance in comparison with matched control subjects whereas performance on other cognitive tests did not differ from the comparison group (see section “Sample Characteristics” Results and **Table 1**).

**Table 1:** Sample characteristics.

	aMCI	Controls	*p*
*n* (females)	26 (6)	20 (7)	0.58
Age (years)	68.5 (4.9)	67.6 (5.1)	0.57
Educationˆ	5.5 (1.1)	5.7 (0.8)	0.66
MMSE	28.6 (1.2)	28.9 (1.1)	0.42
GDS	7.12 (5.8)	2.4 (3.6)	<0.01
GDS non-apathy	5.2 (4.5)	1.6 (2.6)	<0.01
AES	30.5 (10.3)	26.2 (4.6)	0.25
RAVLT-IR	30.8 (8.3)	41.6 (8.3)	<0.01
RAVLT-DR	5.1 (2.4)	8.8 (2.7)	<0.01
BNT	26.7 (2.7)	26.5 (2.9)	0.67
DS-forward	5.8 (1.2)	6.2 (1.3)	0.42
DS-backward	4.7 (0.7)	4.8 (1.0)	0.78
DSS	46.3 (6.9)	50.4 (7.7)	0.09
TMT-A	38.2 (11.9)	39.9 (17.8)	0.63
TMT-B	82.8 (25.2)	83.5 (42.9)	0.78
Stroop test (interference)	56.1 (32.5)	49.4 (14.5)	0.57
Hayling test	13.7 (3.8)	15.0 (4.3)	0.36

*Group comparisons were performed with Chi-square tests for gender, and Student’s T-test or Mann-Whitney U-test for continuous measures. Numbers given are mean (standard deviation) except for sample size and gender. ˆEducation levels are reported according to the Verhage system. MMSE, Mini Mental State Examination; GDS, Geriatric Depression Scale; AES, Apathy Evaluation Scale; RAVLT-IR, Rey’s Auditory Verbal Learning Test – Immediate Recall; RAVLT-DR: Rey’s Auditory Verbal Learning Test –Delayed Recall; BNT, Boston Naming Test; DS: Digit Span Test; DSS, Digit Symbol Substitution Test; TMT, Trail Making Test; aMCI, amnestic Mild Cognitive Impairment.*

### MRI Acquisition

**FIGURE 1:**
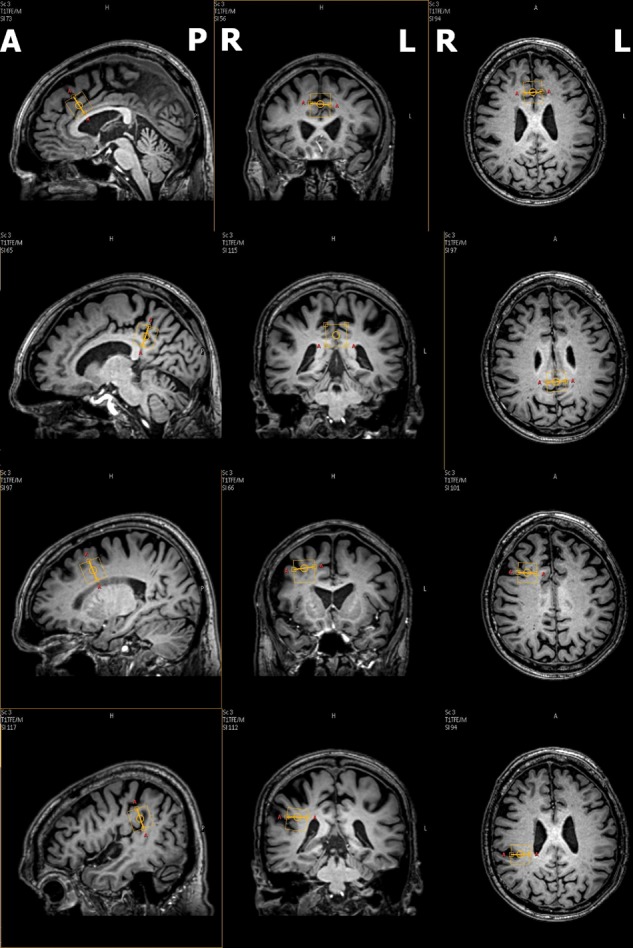
Voxel position on sagittal, coronal and transverse sections. Bounding box of the voxel is shown in yellow. (A: Anterior; P: Posterior; R: Right; L: Left).

Subjects underwent scanning in a 3T Philips Intera MRI scanner (Best, Netherlands) equipped with a 32-channel SENSE head coil. A whole-brain high-resolution anatomical 3D T1-weighted scan was acquired for positioning of ^1^H-MRS voxels and for separation of tissue classes within each spectroscopic voxel [repetition time (TR) 9 ms; echo time (TE) 3.6 ms; flip angle (FA) 8°; field of view (FOV) 256 × 232; 170 slices; voxel size 1 mm × 1 mm × 1 mm]. Spectra were acquired from four locations sequentially, each with a single voxel measuring 20 mm × 20 mm × 20 mm. Following automated first-order B0 shimming, water unsuppressed and chemical shift water suppression (CHESS) spectra were acquired using Point Resolved Spectroscopy (PRESS) sequence with a TR = 2000 ms, TE = 35 ms, 128 signal excitations and 1024 data points. Voxel locations are shown in **Figure 1**. The first voxel was placed in the bilateral DACC, aligned to the callosal sulcus inferiorly and the anterior border of the voxel was located ∼7–10 mm posterior to the genu of the corpus callosum while the superior border extended into the superior frontal gyrus. The second voxel was placed midline in the PCC/inferior precuneus as described for ^1^H-MRS studies in AD (Kantarci et al., 2008). The third voxel was placed in the right DLPFC, with the inferior border aligned with the corpus callosum, and superior to the lateral fissure. The fourth voxel was placed in the right TPC, covering the posterior ascending ramus of the sylvian fissure and surrounding supramarginal gyrus. The third and fourth voxels also covered the underlying WM considerably owing to the size of the voxel and necessity of minimizing CSF within the voxel.

### Analyses

The metabolites NAA, Cho, mI, Cr, and Glx were quantified using LCModel software package (Provencher, 2001). The acquired spectra were considered reliable if Cramer-Rao lower bounds (CRLB), used for estimating model fitting error for each metabolite, were less than 20% (Cavassila et al., 2001). Data not meeting this criterion were excluded from analyses. Final group sizes for each metabolite are given in **Table 2**. Metabolite values were referenced to internal water concentration obtained from a water-unsuppressed^1^H-MRS acquisition (Alger, 2010). In addition, NAA/mI ratios were calculated. Tissue proportions of gray matter, WM, and CSF in each voxel were extracted from T1-weigthed images by masking the ^1^H-MRS voxel position using custom scripts and subsequently segmented using SPM12^1^.

**Table 2:** Partial correlations (Spearman) between apathy scores (AES) and ^1^H-MRS metabolites, controlling for gender, depression scores (GDS non-apathy), and proportion of white matter (WM) and CSF in the voxel.

	N (aMCI_A/aMCI_NA/CN)	*R*	*p* (unc)	*p* (fdr)
**Posterior cingulate cortex (PCC)**				
NAA	46 (8/18/20)	0.015	0.36	0.55
Cho	46 (8/18/20)	–0.39	**0.01**	0.07
mI	45 (8/18/19)	–0.12	0.46	0.55
Cr	46 (8/18/20)	–0.16	0.31	0.55
Glx	46 (8/18/20)	0.05	0.76	0.76
NAA/mI	45 (8/18/19)	0.14	0.37	0.55
**Dorsal anterior cingulate cortex (DACC)**				
NAA	46 (8/18/20)	0.17	0.28	0.85
Cho	46 (8/18/20)	0.01	0.99	0.99
mI	42 (7/17/18)	0.06	0.74	0.99
Cr	46 (8/18/20)	0.06	0.69	0.99
Glx	41 (8/15/18)	0.32	0.05	0.32
NAA/mI	42 (7/17/18)	0.01	0.96	0.99
**Right dorsolateral prefrontal cortex (R-DLPFC)**				
NAA	43 (7/16/20)	–0.04	0.79	0.99
Cho	43 (7/16/20)	–0.37	**0.02**	0.13
mI	38 (7/15/16)	<0.01	0.99	0.99
Cr	43 (7/16/20)	0.08	0.61	0.99
Glx	41 (7/15/19)	0.03	0.88	0.99
NAA/mI	38 (7/15/16)	<0.01	0.99	0.99
**Right temporo-parietal cortex (R-TPC)**				
NAA	45 (8/18/19)	–0.11	0.48	0.48
Cho	45 (8/18/19)	**-0.53**	**0.0003**	**0.002**
mI	45 (8/18/19)	**-0.37**	**0.016**	**0.049**
Cr	45 (8/18/19)	–0.15	0.36	0.44
Glx	45 (8/18/19)	–0.19	0.23	0.35
NAA/mI	45 (8/18/19)	0.28	0.08	0.16

*AES, Apathy Evaluation Scale; GDS, Geriatric Depression Scale; N, sample size; aMCI_A, aMCI with apathy; aMCI_NA, aMCI without apathy; CN, cognitively normal matched controls; NAA, N-acetylaspartate; Cho, Glycerophosphocholine and phosphocholine; mI, myo-inositol; Cr, Creatine and phosphocreatine; Glx, Glutamate and glutamine. Significant values are given in bold. p (unc), uncorrected p-values; p (fdr), false discovery rate corrected p-values.*

Statistical analyses were performed in R^2^. Demographic data between the groups were compared with Mann-Whitney *U*-tests or chi-squared tests (for gender). Significant differences on the omnibus test were further evaluated with the Dunn test. Since GDS scores differed significantly between controls and aMCI patients, we calculated a sub-score (GDS non-apathy) by leaving out six items of the GDS (No. 2, 12, 19, 20, 21, and 28) that were identified as an apathy component in a factor analysis of a large independent sample of healthy older adults (Adams et al., 2004). Spearman correlations between the AES score, and GDS scores were calculated.

Partial correlations (Spearman) between metabolites and AES scores were determined, after controlling for GDS non-apathy score, and proportion of WM and CSF content in the voxel as covariates. As gender differences have been noted in the association between neural correlates and apathy (Spalletta et al., 2013), gender was also included as a covariate. Additionally, analyses of covariance (ANCOVA) were conducted for differences in metabolites between aMCI and control groups. Results were considered significant at FDR-corrected two-tailed *p* < 0.05. *Post hoc* exploratory ANCOVA were conducted for differences between aMCI with apathy, aMCI without apathy, and control group (uncorrected *p*-value reported).

## Results

### Sample Characteristics

Demographic and clinical characteristics of the sample are given in **Table 1**. The patient and control group were comparable except for the GDS score. The GDS non-apathy subscore, denoting depression independent of apathy, was significantly higher in the patient group compared to controls. The AES score was significantly correlated with the GDS total score (*r* = 0.32, *p* = 0.03) but not with the GDS non-apathy score (*r* = 0.23, *p* = 0.13). The GDS non-apathy scores between the MCI with and without clinically-defined apathy did not differ [t = 1.796, *p* = 0.0796 (uncorrected)]. However, both patient subgroups showed higher GDS non-apathy scores than the matched control group (MCI_apathy vs. HC: *t* = 3.610, *p* < 0.001; MCI_non-apathy vs. HC: *t* = 2.299, *p* = 0.026). Including age and education level as covariates, aMCI subjects showed lower performance on the 15-word Rey’s auditory verbal learning test for immediate recall (*t* = -4.32, *p* < 0.001) and delayed recall (*t* = -4.82, *p* < 0.001), whereas their performance on tests for processing speed, confrontational naming, and executive functions did not differ significantly from that of control subjects (**Table 1**).

### Associations Between Apathy and Neurometabolites

**FIGURE 2:**
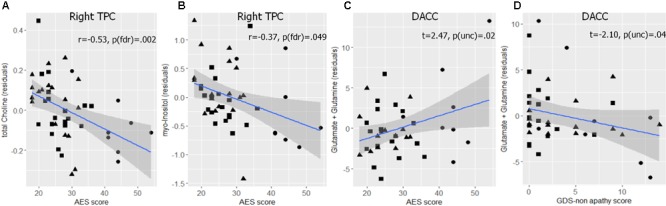
Associations between metabolites, and apathy and depression scores. In all figures, • represent amnestic mild cognitive impairment (aMCI) with apathy group, ▲ represent aMCI without apathy group, and ■ represent matched cognitively normal control group. **(A)** shows a decline in glycerophosphocholine and phosphocholine (Cho) levels and **(B)** shows a decline in myo-inositol (mI) levels with increasing apathy in the right temporo-parietal cortex (TPC). **(C)** shows increasing glutamate and glutamine (Glx) with apathy scores and **(D)** shows decreasing Glx with increasing depression [Geriatric Depression Scale (GDS) score without apathy items] in the dorsal anterior cingulate cortex (DACC).

Partial correlations between metabolite levels and AES scores controlled for proportion of WM and CSF in the voxel, gender, and GDS non-apathy score, showed that higher AES scores were correlated with lower Cho (*r* = -0.53, *p_fdr_* = 0.002, *n* = 45) and lower mI (*r* = -0.42, *p_fdr_* = 0.016, *n* = 45) in the right TPC. Uncorrected for multiple comparisons, there were also correlations seen between AES score and lower Cho (r = -0.37, *p_unc_* = 0.015, *p_fdr_* = 0.092, *n* = 45) in the PCC and higher NAA/mI (*r* = 0.33, *p_unc_* = 0.033, *p_fdr_* = 0.07, *n* = 45) in the right TPC. These results did not survive correction for multiple comparisons. No other significant associations were found between any metabolites and AES score in the assessed brain regions (**Table 2** and **Figure 2**).

The analyses also did not show neurometabolite differences in any region between the aMCI and control group (**Table 3**). Notably, the NAA/mI ratio in the PCC did not differ significantly between the two groups. In addition, given that apathy increases the risk of progression to AD, we expected to find an association between AES scores and metabolite levels in the PCC. As this was not the case, we conducted an exploratory ANCOVA to determine if presence or absence of apathy was associated with different metabolite levels in the four voxels of interest. The groups, defined according to clinical diagnosis, were aMCI with apathy, aMCI without apathy, and controls. We included the following variables as covariates: gender, GDS non-apathy score, and WM and CSF proportions.

**Table 3:** Differences in metabolite levels between aMCI and cognitively normal controls.

	PCC	DACC	R-DLPFC	R-TPC
NAA	1.64 (5,40), *p* = 0.17	**6.50 (5,40), *p* < 0.01**	1.41 (5,38), *p* = 0.24	0.21 (5,39), *p* = 0.95
		*t* = 0.93, *p* = 0.36		
Cho	1.00 (5,40), *p* = 0.43	0.23 (5,40), *p* = 0.95	1.26 (5,38), *p* = 0.30	0.93 (5,39), *p* = 0.47
mI	0.92 (5,39), *p* = 0.48	0.16 (5,36), *p* = 0.97	0.86 (5,32), *p* = 0.52	1.44 (5,39), *p* = 0.23
Cr	**3.38 (5,40), *p* = 0.01**	0.29 (5,40), *p* = 0.91	1.11 (5,38), *p* = 0.37	0.72 (5,39), *p* = 0.61
	t = -1.78, *p* = 0.08			
Glx	0.63 (5,40), *p* = 0.68	1.57 (5,35), *p* = 0.19	0.86 (5,36), *p* = 0.52	0.41 (5,39), *p* = 0.84
NAA/mI	1.98 (5,39), *p* = 0.10	0.28 (5,36), *p* = 0.92	0.72 (5,32), *p* = 0.61	1.09 (5,39), *p* = 0.38

*Numbers given are the value of F-test of ANCOVA for group differences and (uncorrected) significance values with fraction of CSF and WM, gender, and Geriatric Depression Scale (GDS) non-apathy score as covariates. When significant, the value of the t-test for difference between aMCI and control groups is given along with significance in bold. aMCI, amnestic mild cognitive impairment; NAA, N-acetylaspartate; Cho, glycerophosphocholine and phosphocholine; mI, myo-inositol; Cr, creatine and phosphocreatine; Glx, glutamate and glutamine; PCC, posterior cingulate cortex; .DACC, dorsal anterior cingulate cortex; R-DLPFC, right dorsolateral prefrontal cortex; R-TPC, right temporo-parietal cortex.*

### Exploratory Analyses

In the exploratory analyses, NAA/mI values were significantly different between the groups [*F*(5,39) = 2.47, *p* < 0.05, adj. *R*^2^ = 0.14]. *Post hoc* testing indicated that the aMCI without apathy group (*n* = 18) showed significantly lower NAA/mI ratio (*t* = -2.2, *p_unc_* = 0.034) as compared to controls (*n* = 20) (Supplementary Table 1). In the DACC, Glx levels between the groups differed significantly [*F*(5,35) = 3.03, *p* = 0.02, adj. *R*^2^ = 0.20] with *post hoc* testing indicating higher Glx levels in the aMCI with apathy group (*n* = 8) (*t* = 2.6, *p_unc_* = 0.013) compared to controls (*n* = 18). In other words, only patients with MCI and apathy, but not those without apathy, show increased Glx as compared to the matched healthy control group without apathy. In the same model, GDS non-apathy scores were associated with lower Glx (*t* = -2.2, *p_unc_* = 0.035).

## Discussion

This study aimed to determine the associations between apathy in aMCI and metabolite levels in four brain regions that have been hypothesized or previously reported to be associated with apathy in aMCI/AD patients. Results showed that greater apathy is associated with reduced Cho and mI in the right TPC in clinically-diagnosed aMCI patients and matched control subjects. No other associations were found. Metabolite levels between the aMCI and control groups were also not significantly different. Because depression scores were included as a covariate, these neural changes can be considered to be independent of possible comorbid depression. Exploratory analyses suggested that reduced NAA/mI as compared to controls, a consistent finding in previous studies in MCI/AD (Adalsteinsson et al., 2000; García Santos et al., 2008; Kantarci et al., 2013; Tumati et al., 2013), was present only in aMCI without apathy group. Overall, our findings (i) support the association between apathy in aMCI and neural changes in the right TPC, (ii) suggest that the mechanisms for this association involve changes in membrane structure (reduced Cho) and glial function (reduced mI), and (iii) indicate that neural changes in aMCI patients with and without apathy may be distinct.

### Neural Mechanisms of Apathy

Apathy develops in a substantial proportion of patients with MCI (Peters et al., 2012). Due to its influence on the disease course and lack of adequate treatments, renewed emphasis has been laid on understanding its neural mechanisms (Lanctôt et al., 2016). Previous studies have linked atrophy, reduced functional connectivity, and reduced metabolism in the TPC with apathy (Donovan et al., 2014; Munro et al., 2015; Gatchel et al., 2017). Findings from the current study support this association between changes in TPC in aMCI patients and apathy. The DACC and adjacent medial frontal regions, previously reported to be associated with apathy (Marshall et al., 2007) showed only a weak association in our exploratory analyses. Furthermore, apathy scores were not correlated with metabolite levels in the DLPFC, which is hypothesized to mediate the cognitive subtype of apathy (Levy and Dubois, 2006), or the PCC, in which consistent changes in metabolite levels in AD/MCI patients were reported in previous studies. Thus, in this first ^1^H-MRS study of all cortical regions implicated in apathy, only neural changes in the TPC were found to have a moderate-to-strong association with apathy in aMCI.

The reduction in Cho and mI levels in association with apathy may have resulted from atrophy in the TPC. This interpretation is supported by studies that report atrophy in the TPC to be a consistent feature in AD patients (Chételat et al., 2005), and have also specifically associated atrophy in this region with apathy symptoms (Donovan et al., 2014), However, as the CSF and WM content in the voxels were controlled for in the analyses, this explanation for the results may be less likely. Moreover, the concentration of NAA in this region, which indicates neuronal viability, was also not reduced as compared to the control group or correlated with the apathy score. Another possibility may be that lower Cho levels are indicative of reduced cholinergic neurotransmission (Duarte et al., 2012), This interpretation is in line with pharmacological studies where drugs that increase the availability of acetylcholine were found to be beneficial for apathy in AD (Berman et al., 2012). However, not all studies find such an effect (Harrison et al., 2016). The lack of unequivocal findings may be attributed to study limitations such as apathy commonly being a secondary outcome measure and assessed with the neuropsychiatric inventory (Harrison et al., 2016), which has only a single item for assessing apathy (Cummings et al., 1994). Nevertheless, atrophy of the nucleus basalis of Meynert, which provides cholinergic innervation to the entire cortex, is known to occur early in the course of AD (Bartus et al., 1982; Grothe et al., 2012). However, it is not clear if this atrophy is associated with specific behavioral symptoms of AD. Taken together, multiple lines of evidence suggest cholinergic dysfunction as a putative mechanism for apathy that should be evaluated especially in the early stages of AD.

### Early Apathy: A Distinct Mechanism?

The ratio of NAA/mI in the PCC on ^1^H-MRS is a proposed biomarker for the diagnoses of AD as it is found to be reduced in AD, MCI, and high risk cognitively normal older adults, as compared with matched controls (Kantarci et al., 2013). Moreover, this ratio was found to gradually decline with disease progression, and was also associated with neuropathological and CSF features of AD (Kantarci et al., 2008; Gomar et al., 2014; Murray et al., 2014). Although based on limited evidence, the TPC in AD may incur changes on ^1^H-MRS similar to that in the PCC (Bittner et al., 2013). However, in the current study, the ratio of NAA/mI in the PCC as well as in the TPC was neither significantly lower in aMCI patients as compared to controls nor was it associated with apathy. Only patients with aMCI without apathy were found to have a lower NAA/mI ratio in the PCC (Supplementary Table 1). This suggests that in those with aMCI and apathy, different neural mechanisms may be affected than in aMCI or AD. Future research should elucidate this relationship between impairments in cognition and behavior.

Clinically-diagnosed aMCI is considered to be a precursor stage for AD, though the broad criteria may result in considerable heterogeneity in terms of progression to AD, and indeed a substantial number of cases do not progress to AD (Mitchell and Shiri-Feshki, 2009). In cognitively normal older adults, development of apathy confers an increased risk of developing AD (Geda et al., 2014). Our results raise the possibility that such individuals may harbor neural changes in the TPC. In such cohorts, the underlying neuropathology may also differ (Saito et al., 2015). Toledo et al. (2013) found that in those with clinical diagnosis of AD or MCI, AD pathology was coincidental in about 40% of subjects with other pathologies such as dementia with Lewy bodies, hippocampal sclerosis, TDP-43 proteinopathy, argyrophilic grain disease and vascular pathology. Similar findings were also reported in independent cohorts (White et al., 2016). In light of these reports, associations between apathy and metabolites in the TPC but not in the PCC suggest that apathy may emerge from pathologies other than those strictly limited to AD proper. This interpretation would imply that in those with aMCI and apathy, memory decline occurs due to impaired TPC function while PCC function remains intact. These suggestions need to be evaluated in larger studies.

### Assessment of Apathy in aMCI

Our results also suggest that detailed evaluation of neuropsychiatric symptoms (NPS) like apathy and depression is of value. The co-morbidity of depression vis-à-vis apathy and their differential influence on the progression of AD has been previously reported (van Reekum et al., 2005; Vicini Chilovi et al., 2009; Palmer et al., 2010; Richard et al., 2012). Studies have also suggested that the neural correlates of the two syndromes may differ (Zahodne et al., 2013; Hollocks et al., 2015). In the current study, the AES and GDS scores were found to be correlated, and this correlation was reduced after excluding items on the GDS that were previously found to be related to apathy. Moreover, the exploratory analyses, while performed in a small sample, suggested that apathy and depression may be associated with divergent changes in Glx levels in the DACC (**Figures 2C,D**). Although this result needs to be replicated in a larger sample, it suggests that the DACC may be involved in apathy as well as depression. This finding provides a possible basis for overlapping brain changes in the two behavioral syndromes. Furthermore, the results also suggest that apathy may emerge from multiple routes (DACC and TPC) in AD. Assessments with dimensional scales will be useful for analyzing links between the subtypes of apathy and neural mechanisms.

As NPS have a substantial influence on disease progression, functional abilities, and need for care, understanding the neural basis of individual NPS is required. The above findings demonstrate that detailed assessments of NPS contribute toward understanding the neural changes in older adults with memory deficits.

### Limitations

While interpreting the results, the limitations of this study need to be considered. First, CSF or PET markers of AD were not available in our sample of aMCI subjects. As a result, we could not examine if the aMCI with and without apathy groups also had different CSF and PET biomarker profiles. Such data could be used to evaluate our interpretation that the two groups harbored distinct neural changes. While deficits limited to memory function like in the current cohort are likely to progress to AD, other outcomes such as development of frontotemporal dementia and diffuse lewy body dementia, stable aMCI (i.e., without further worsening) or even reversion to normal cognitive function may occur (Mitchell and Shiri-Feshki, 2009) and need to be considered when interpreting the results.

Second, cognitive function was not included as a covariate in the analyses. Because apathy becomes more common and severe from single domain aMCI to multi-domain MCI (Di Iulio et al., 2010), it may suggest that cognitive dysfunction and apathy either share a common neural substrate or their underlying brain changes occur independently but in parallel. From the current analyses, it cannot be said if the metabolite changes associated with apathy are independent of cognitive deficits. The largely intact cognitive function in the study subjects limits exploration of this possibility. Moreover, past studies suggest that cognitive and behavioral impairments may be distinct components of dementia (Spalletta et al., 2004; Ismail et al., 2016). Third, the sample size of the aMCI with apathy group was relatively small. However, the incidence of apathy in very early stages of MCI (between 5 and 15%) makes inclusion of large samples with apathy difficult (Peters et al., 2012). Lastly, spectra were only acquired from the right side of the brain for the DLPFC and TPC. Therefore, lateralization of brain changes in relation to apathy could not be evaluated. Despite these limitations, this first MRS study of apathy in aMCI patients has provided novel findings regarding the neural mechanisms of apathy. Furthermore, apathy was assessed using the AES in cohort with relatively intact cognitive function, minimizing the likelihood of other comorbid symptoms. This study also examined metabolite changes in all regions hypothesized to be associated with apathy.

## Conclusion

In a clinically-diagnosed aMCI cohort, preliminary evidence show that apathy symptoms may be suggestive of distinct neural changes from those in aMCI without apathy. The TPC may be a key region for the neural correlates of apathy as indicated by reduced Cho and mI levels indicative of alterations in membranal and glial function. Our tentative finding suggesting different glutamatergic changes in the DACC for apathy versus depression needs further investigation. MRS at higher field strengths (e.g., 7T) may also yield additional insights into neurometabolite correlates of apathy.

## Author Contributions

ST designed the study, analyzed the data, interpreted the results, and wrote the manuscript. EO and FR recruited and assessed study subjects and critically revised the manuscript. J-BM contributed to the analysis, interpretation, and revision of manuscript. SM interpreted the results and critically revised the manuscript. PDD contributed to study design, subject recruitment, interpretation of results, and critical revision of manuscript. AA designed and supervised the study, interpreted the results, and critically revised the manuscript.

## Conflict of Interest Statement

The authors declare that the research was conducted in the absence of any commercial or financial relationships that could be construed as a potential conflict of interest.

## Footnotes

1 http://www.fil.ion.ucl.ac.uk/

2 https://www.r-project.org/

## Abbreviations

H-MRS: proton magnetic resonance spectroscopy
AD: Alzheimer’s disease
AES: Apathy Evaluation Scale
aMCI: amnestic mild cognitive impairment
Cho: glycerophosphocholine and phosphocholine
Cr: creatine and phosphocreatine
DACC: dorsal anterior cingulate cortex
DLPFC: dorsolateral prefrontal cortex
fdr: false discovery rate
GDS: Geriatric Depression Scale
Glx: glutamate and glutamine
mI: myo-inositol
MMSE: mini mental state examination
NAA: N-acetylaspartate
PCC: posterior cingulate cortex
TPC: temporo-parietal cortex
WM: white matter
