# Supplementary material for: Lower Choline and Myo-Inositol in Temporo-Parietal Cortex Is Associated With Apathy in Amnestic MCI
Source: Front Aging Neurosci. 2018 Apr 13;10:106. doi: 10.3389/fnagi.2018.00106 (PMC5909116; doi:10.3389/fnagi.2018.00106)
Supplement: Supplementary file 1 [file Table_1.docx]

**Supplementary Table: Results of exploratory analyses of metabolites in each regions and diagnostic groups, gender, depression scores (GDS-non apathy scores), and proportion of white matter and CSF in the voxel. Table shows overall test for model (F (df), *p*) and unstandardized coefficients (β), t-values and significance values for variables of interest.**

|  |  |  | **aMCI with apathy vs controls** | | | **aMCI without apathy vs controls** | | | **GDS-non apathy** | | | **Gender** | | |
| --- | --- | --- | --- | --- | --- | --- | --- | --- | --- | --- | --- | --- | --- | --- |
|  | **F (df), *p*** | **Intercept** | **β** | **t** | ***p*** | **β** | **t** | ***p*** | **β** | **t** | ***p*** | **β** | **t** | ***p*** |
| **Posterior Cingulate Cortex** | | | | | | | | | | | | | | |
| **NAA** | 1.46 (6,39), *p*=.22 | 8.84 | -0.05 | -0.14 | .89 | -0.28 | -1.30 | .20 | 0.03 | 1.19 | .24 | 0.46 | 1.98 | .05 |
| **Cho** | 0.88 (6,39), *p*=.52 | 1.49 | -0.09 | -1.09 | .28 | -0.04 | -0.78 | .44 | 0.01 | 1.13 | .27 | 0.01 | 0.20 | .84 |
| **mI** | 1.11 (6,38), *p*=.37 | 5.64 | -0.14 | -0.49 | .63 | 0.24 | 1.13 | .26 | -0.03 | -1.10 | .30 | -0.09 | -0.39 | .70 |
| **Cr** | 2.75 (6,39), *p*=.03 | 7.13 | -0.22 | -1.08 | .29 | -0.24 | -1.73 | .09 | **0.05** | **2.84** | **<.01^*^** | 0.10 | 0.70 | .49 |
| **Glx** | 0.54 (6,39), *p*=.77 | 10.76 | 0.29 | 0.40 | .69 | <0.01 | 0.01 | .99 | 0.03 | 0.63 | .53 | 0.60 | 1.14 | .26 |
| **NAA/mI** | 2.57 (6,38), *p*=.03 | 1.66 | 0.05 | 0.43 | .67 | **-0.18** | **-2.21** | **.03^*^** | 0.02 | 2.09 | .04 | 0.14 | 1.59 | .12 |
| **Dorsal anterior cingulate cortex** | | | | | | | | | | | | | | |
| **NAA** | 5.38 (6,39), *p*<.01 | 8.86 | 0.43 | 1.03 | .31 | 0.21 | 0.76 | .45 | -0.02 | -0.53 | .60 | 0.27 | 0.91 | .37 |
| **Cho** | 0.35 (6,39), *p*=.90 | 2.03 | -0.10 | -0.37 | .71 | 0.14 | 0.84 | .41 | -0.01 | -0.33 | .74 | -0.05 | -0.30 | .77 |
| **mI** | 0.16 (6,35), *p*=.99 | 5.67 | 0.37 | 0.26 | .80 | 0.86 | 0.94 | .36 | -0.03 | -0.28 | .78 | <-0.01 | -0.01 | .99 |
| **Cr** | 0.53 (6,39), *p*=.78 | 6.75 | -0.30 | -0.39 | .70 | 0.63 | 1.26 | .22 | -0.01 | -0.20 | .84 | -0.28 | -0.52 | .61 |
| **Glx** | 2.49 (6,34), *p*=.04 | 17.20 | **4.94** | **2.47** | **.02^*^** | 0.30 | 0.23 | .82 | **-0.34** | **-2.10** | **.04^*^** | -0.59 | -0.43 | .67 |
| **NAA/mI** | 0.25 (6,35), *p*=.96 | 1.43 | -0.14 | -0.49 | .63 | -0.04 | -0.21 | .83 | 0.01 | 0.49 | .63 | 0.10 | 0.51 | .61 |
| **Right dorsolateral prefrontal cortex** | | | | | | | | | | | | | | |
| **NAA** | 1.28 (6,37), *p*=.29 | 11.41 | -0.41 | -0.55 | .59 | 0.18 | 0.35 | .73 | -0.10 | -1.59 | .12 | 0.23 | 0.46 | .65 |
| **Cho** | 1.26 (6,37), *p*=.30 | 1.90 | -0.31 | -2.09 | .04 | -0.16 | -1.53 | .14 | 0.01 | 0.87 | .39 | -0.12 | -1.20 | .24 |
| **mI** | 0.74 (6,31), *p*=.62 | 3.61 | -0.31 | -0.53 | .60 | -0.56 | -1.53 | .14 | -0.02 | -0.31 | .76 | 0.01 | 0.03 | .98 |
| **Cr** | 1.01 (6,37), *p*=.43 | 7.61 | -0.69 | -1.67 | .10 | -0.39 | -1.40 | .17 | <0.01 | 0.07 | .95 | 0.05 | 0.19 | .85 |
| **Glx** | 0.78 (6,35), *p*=.59 | 7.81 | 0.33 | 0.28 | .79 | -0.45 | -0.58 | .58 | 0.12 | 1.22 | .23 | -1.03 | -1.31 | .20 |
| **NAA/mI** | 0.60 (6,31), *p*=.73 | 2.85 | 0.20 | 0.69 | .50 | 0.27 | 1.46 | .16 | <-0.01 | 0.19 | .85 | 0.01 | 0.06 | .95 |
| **Right temporo-parietal cortex** | | | | | | | | | | | | | | |
| **NAA** | 0.31 (6,38), *p*=.93 | 8.70 | -0.13 | -0.42 | .68 | 0.12 | 0.54 | .59 | <-0.01 | 0.02 | .98 | <-0.01 | -0.04 | .97 |
| **Cho** | 1.40 (6,38), *p*=.24 | 1.40 | -0.11 | -1.39 | .17 | 0.03 | 0.47 | .64 | <0.01 | 0.48 | .63 | <0.01 | <.01 | .99 |
| **mI** | 1.80 (6,38), *p*=.12 | 5.20 | -0.22 | 0.76 | .45 | 0.27 | 1.23 | .23 | -0.05 | -1.89 | .07 | 0.14 | .69 | .49 |
| **Cr** | 1.18 (6,38), *p*=.34 | 7.65 | -0.43 | -1.60 | .12 | 0.03 | 0.15 | .89 | 0.01 | 0.48 | .63 | -0.05 | -0.27 | .79 |
| **Glx** | 0.54 (6,38), *p*=.77 | 11.39 | -0.31 | -0.62 | .54 | 0.20 | 0.53 | .60 | 0.01 | 0.25 | .81 | -0.48 | -1.40 | .17 |
| **NAA/mI** | 1.19 (6,38), *p*=.338 | 1.46 | 0.11 | 0.67 | .50 | -0.09 | -0.68 | .50 | 0.02 | 1.59 | .12 | -0.04 | -0.38 | .71 |

NAA: N-acetylasparte; Cho: Glycerophosphocholine & phosphocholine; mI: myo-inositol; Cr: Creatine & phosphorcreatine; Glx: Glutamate & glutamine.
